# Dry-Cured Ham, ‘*Kraški Pršut*’, from Heavy Pig Production—A Pilot Study Focusing on the Effect of Ham Weight and Salting

**DOI:** 10.3390/foods13223620

**Published:** 2024-11-13

**Authors:** Bojana Savić, Marjeta Čandek-Potokar, Martin Škrlep

**Affiliations:** 1 Agricultural Institute of Slovenia, Hacquetova Ulica 17, 1000 Ljubljana, Slovenia; bojana.savic@kis.si (B.S.); meta.candek-potokar@kis.si (M.Č.-P.); 2 Faculty of Agriculture and Life Sciences, University of Maribor, Pivola 10, 2311 Hoče, Slovenia

**Keywords:** ham weight, reduced salt, dry-cured ham quality, sensory analysis, CATA

## Abstract

A pilot study was conducted with the aim of adapting the processing of “*Kraški pršut*”, dry-cured ham, for thighs from heavy pigs. The focus was on the effect of ham weight and salting duration on the quality of dry-cured ham. From a pool of thighs harvested from heavy pigs, a total of 32 green hams were selected (from 16 carcasses) based on weight (two classes; L—lighter, H—heavier) and we used left and right ham for either the standard or a shortened salting phase. Salting duration consisted of phase 1 (7 days for all hams) and phase 2 (7 or 14 days for L, 10 or 17 days for H, in the case of shortened and standard salting, respectively). Equivalent conditions for all hams were maintained during the remaining phases, with a total maturation period of 18 months. The analysis focused on chemical, physical and rheological properties, sensory attributes, and consumer perceptions. The H hams had lower processing losses, resulting in higher moisture and water activity, lower salt content in internal *biceps femoris* muscle, and a softer texture (instrumental and sensory) than L hams. The salting duration mainly affected weight losses in the salting phase and, consequently, salt content, which was lower in the shortened salting phase, while no effects were observed on texture. The sensory panel perceived weight’s effect on hardness, with L hams being perceived as harder, and salting’s effect on sourness, with hams submitted to longer salting perceived as sourer than H hams. Consumer testing indicated a general preference for softer and less salty hams. Overall, the results show that the applied reduction in salting duration was not substantial, and future trials should explore further optimization in terms of salting and resting phases.

## 1. Introduction

“*Kraški pršut*” belongs to the family of a Mediterranean type of dry-cured hams and holds Protected Geographical Indication (PGI) status under EU legislation [1]. The production process is characterized by dry salting (using only sea salt), the absence of smoking, and a long maturation period (at least 12 months). It is the most valued dry-cured product by Slovenian consumers [2]. The PGI designation for “*Kraški pršut*” does not affect the origin of the hams, meaning that while the processing is tied to a specific region, it allows for flexibility regarding the sourcing of the raw materials. Currently, producers of “*Kraški pršut*” are sourcing raw hams from standard pig fatteners, but they aim to shift towards developing a higher-quality niche product, which also includes reducing saltiness in the final product.

The quality of the raw material (i.e., fresh hams) has high importance in dry-cured ham processing and is affected by many factors [3]. To produce a high-quality product, it is beneficial to select heavier thighs from mature pigs that have a greater proportion of subcutaneous and intramuscular fat [4]. For instance, Italian PDO ham specifications mandate that fresh hams must come from heavy pigs (>160 kg) at least 9 months old, of approved genetic lines and adequate fat quantity and quality. The PGI for “*Kraški pršut*” does not mandate the use of specific pig genotype or weight, meaning that hams from conventional pig production are commonly used. Such hams are of lower weight and very muscled and lean, which is generally associated with lower sensory quality of dry-cured ham [5].

Reducing the level of salt content of dry-cured ham is challenging since many factors, i.e., weight, pH, and fat content, affect salt uptake. Salt plays a crucial role in dry-curing, influencing various aspects such as safety and taste [6]. However, excessive salt intake is problematic for health, e.g., causing hypertension [7], and consumers are therefore increasingly demanding products with lower sodium content [8]. Considering the essential functions of salt in meat products, like safety, its reduction must be carefully implemented [9]. In addition, salt reduction may be associated with increased proteolysis and cause undesirable changes in texture and ham aroma [10,11]. To ensure a consistent saltiness of the product, dry-cured ham processors usually categorize hams by weight and determine the salting duration based on the guideline of one day of salting for each kilogram of green ham. However, weight-based salting time neglects other factors like fat content or meat pH, which also influence salt absorption [12].

Different aspects of “*Kraški pršut*” quality have been examined before [10,13,14,15]. However, the studies were conducted on standard products and no research on “*Kraški pršut*” has so far made a thorough investigation of hams sourced from heavy pig production. In addition, as the producers want to develop an innovative product with heavy hams, process optimization is needed, particularly in the adaptation of the salting phase to heavier hams. Given this context, the present study aimed to investigate the physicochemical properties, instrumental texture, and sensory characteristics of “*Kraški pršut*” considering two weight classes and two salting durations. Additionally, it sought to evaluate consumer acceptability of this innovation.

## 2. Materials and Methods

### 2.1. Raw Material and Ham Processing

**Figure 1:**
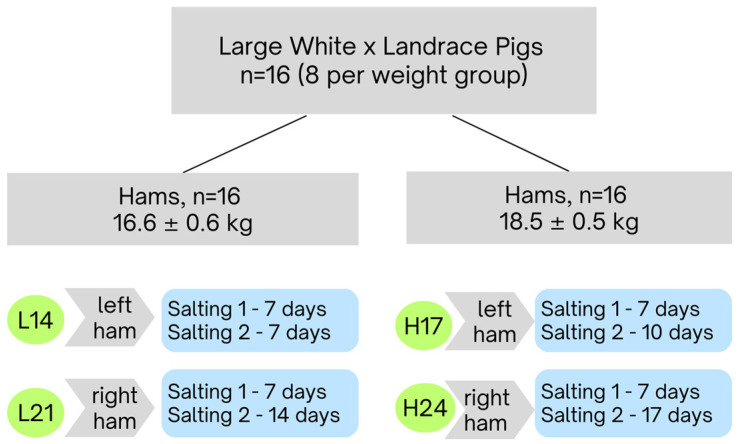
Experimental design.

A local pig producer performed a pilot fattening of crossbred castrated pigs (crosses between landrace and large white) to a heavy weight (>160 kg) for the dry-cured ham producer. At the slaughter line, the carcasses that were free of bruises were pre-selected for dry-cured ham processing and their thighs were labeled with an identification mark. The next day, the thighs were trimmed and shaped to the prescribed form and measurements were taken of green ham weight, subcutaneous fat thickness, and pH value in the *semimembranosus* (SM) muscle. Subsequently, 32 hams (both left and right hams from 16 carcasses) were randomly selected for further processing, so as to have two weight classes that differed by 2 kg; L (lighter hams, 16.6 ± 0.6 kg) and H (heavier hams, 18.5 ± 0.5 kg). Hams from each weight group underwent two different salting durations, with the right hams assigned to shorter and the left hams assigned to longer salting periods. The salting duration was adapted to the weight of the hams, being typically 1 day per kilogram of the green ham [16]. The experimental design is graphically illustrated in Figure 1. All hams were submitted to two-phase salting (at 2–4 °C). The initial phase lasted for 7 days, regardless of ham weight. Subsequently, the second salting phase was different in short and standard salting but at the same time adapted to weight: thus, L hams were salted for either 14 days (L14) or 21 days (L21), while H hams were salted for either 17 days (H17) or 24 days (H24). On average, 0.6 kg of salt was applied at first salting and 0.4 kg was applied at second salting. The salt was applied in excess (see Supplementary Material Table S1). After the salting phase, the remaining salt was brushed away; hams were washed and left to rest at 4–6 °C and 70–85% relative humidity (RH). On the 105th day of processing, the hams were submitted to drying and ripening (14–20 °C, 60–80% RH) until reaching the prescribed weight loss (>33%). During this phase, after reaching 25% weight loss, the open area of hams was covered with a mixture of fat, rice flour, and white pepper to prevent over-desiccation and crusting. The overall processing duration was 600 days.

### 2.2. Physical–Chemical Measurements

At the end of processing, the samples were taken from the middle part of the dry-cured hams (for details, see Škrlep et al. [17]), which is composed of SM, *biceps femoris* (BF) and *semitendinosus* (ST) muscles, and color parameters (CIE L*, a*, b*) were measured on-the-spot with a Minolta Chroma Meter CR-400 (Minolta Co., Ltd., Osaka, Japan) applying the standard illuminant, D65, the standard observed angle of 2°, and an 11 mm aperture calibrated against a white tile (Y = 85.5; x = 0.3203; y = 0.3381). The color evaluation was performed in triplicate on the freshly cut surfaces of dry-cured ham muscles (SM, BF, and ST) and subcutaneous fat layer. Thereafter, the samples were taken to the laboratory where they were further divided into three portions: (a) for chemical composition, (b) for instrumental texture measurements, and (c) for sensory analysis. Water, intramuscular fat (IMF), salt, and protein content were determined using near-infrared spectroscopy (NIR) with an NIR Systems 6500 Monochromator (Foss NIR System, Silver Spring, MD, USA) (see Supplementary Material Table S2 for calibration parameters). Total nitrogen content and non-protein nitrogen (NPN) content were determined according to Škrlep et al. [17]. The proteolysis index (PI) was calculated as the ratio of non-protein nitrogen to total nitrogen, expressed as a percentage. Water activity (a_w_) was assessed using an Aqua LAB 4TE apparatus (Decagon Devices Inc., Pullman, WA, USA). Lipid oxidation of dry-cured ham muscles was evaluated by measuring thiobarbituric acid reactive substances (TBARSs) following the method described by Marušić Radovčić et al. [18].

### 2.3. Texture Measurements

Instrumental texture measurements were performed on the SM, BF, and ST muscles, using texture profile analysis (TPA) and a stress relaxation (SR) test as detailed in our previous study [17]. The tests were performed with a texture analyzer (Ametek Lloyd Instruments, Ltd., Bognor Regis, UK) equipped with a 50 kg load cell and a 50 mm diameter compression plate. In the SR test, samples were compressed perpendicular to the fiber bundle direction for 25% of their initial height at a crosshead speed of 1 mm/s. For the TPA test, samples underwent two compression cycles to 50% of their original height, also at a crosshead speed of 1 mm/s. The following TPA characteristics relevant for solids were determined: hardness (N), cohesiveness (dimensionless), springiness (mm), chewiness (N*mm), and adhesiveness (N*mm).

### 2.4. Sensory and Consumer Analysis

The sensory evaluation was carried out by an accredited laboratory with a panel of 8 certified assessors all trained according to ISO 11132:2012 standards [19]. The assessment of the whole slice of dry-cured ham (keeping 1 cm of subcutaneous fat) was made evaluating the following 16 attributes: fresh meat odor, spiciness, herbal notes, muscle and fat color intensity, surface moisture, color uniformity, visible intramuscular fat (marbling), saltiness, sweetness, sourness, bitterness, hardness, solubility, and pastiness. Each attribute was evaluated on a 10 cm unstructured linear scale anchored at both ends, with 0 representing no perception and 10 indicating maximum intensity. Additionally, a hedonic sensory test was conducted with 95 consumers (43 males and 50 females, with an average age of 48 years). Participants were asked to (i) rate their liking of the products on a 5-point Likert scale, ranging from “strongly dislike” to “strongly like” with a neutral option of “neither like nor dislike”, and (ii) to indicate the sensations experienced by using a “check all that apply” (CATA) method [20]. Consumers were asked to mark the descriptors they perceived as applicable to the product. There were 16 descriptors included in the CATA method, namely, hard, soft, soluble, crumbly, dry, raw, mature, salty, non-salty, sweet, fatty, lean, juicy, aromatic, tasty, and unpleasant taste. The descriptors were selected and agreed on in collaboration with the personnel from the dry-cured ham consortium.

### 2.5. Statistical Analysis

The statistical analysis was conducted using SPSS version 28.0.0 (IBM Corp., Armonk, NY, USA, 2021). Fischer’s Exact test was used to compare the results of consumer testing with CATA. As for all other data measurements, they were analyzed using a linear mixed model with the fixed effects of weight group and salting time (nested within the weight group). The mean values of treatment groups were compared using a *t*-test. The level of significance was considered at a *p*-value < 0.05.

## 3. Results and Discussion

### 3.1. Raw Material (Green Ham) Traits and Ham Processing Losses

The data in Table 1 show that at the start of the processing the green hams of different weight classes were similar in terms of the pH value of the SM muscle, while there was a slight difference in fat cover thickness, namely that heavier hams had, on average, 3 mm thicker subcutaneous fat cover.

Lower processing losses were observed for H than L hams (Table 2). The effect of weight was significant in the first salting phase, which was equally long (7 days) in all groups, at the end of resting period and at the end of processing. On the other hand, processing loss in the second salting phase was affected by salting duration and was higher in groups with 7 days more exposure to salt. Overall, the observed difference in processing loss of 1.6% was mainly due to the initial phase (1.4% difference after resting) and could be related to subcutaneous fat cover, which was slightly thicker (3 mm), as previously suggested [21].

### 3.2. Physical–Chemical Measurements of Dry-Cured Ham Muscles

#### 3.2.1. Chemical Traits of Dry-Cured Hams

Green ham weight and salting duration differences were observed in the chemical traits of the dry-cured hams (Table 3). Heavier hams had higher water content and water activity and lower protein content in both muscles, which is consistent with their lower processing losses. Similarly, BF muscle salt content was lower in heavier hams, which corroborates with processing loss, as salt content is negatively correlated with this parameter [22].

A shorter salting duration resulted in a lower salt content in both the SM and BF, with the reduction being statistically significant (*p* < 0.05) in L hams, and a tendency towards significance (*p* < 0.10) in H hams. The salting time reduction of 7 days resulted in generally small differences in the salt concentration of the final product. This can be explained with the asymptotic function of salt uptake [23] and shows that to achieve a meaningful salt reduction, the salting phase was not sufficiently shortened. Despite the reduced salt content, which hinders proteolytic enzymes and protein breakdown [10,24,25], a lower proteolysis index was observed only in L hams (*p* < 0.05), while the difference was not sufficient to affect the proteolysis index in H hams. Regarding the oxidative stability, no significant effect of green ham weight or salting duration was observed on TBARS values. In general, salt is considered a pro-oxidative agent in muscle foods [26,27], promoting lipid oxidation. However, in dry-cured ham the pro-oxidative effects are limited or unclear, as shown, for instance, by Andrés et al. [28,29].

#### 3.2.2. Color of Dry-Cured Hams

To assess the impact of weight and salting duration on the color of dry-cured ham, the measured color parameters L*, a*, and b* (see Supplementary Material Table S3) were combined into a single numerical value, E*ab, which is considered as an indicator of the amount of color difference [30]. Some individual color parameters were significantly affected by ham weight or salting. Results like higher SM lightness (L*) in H compared to L hams can be attributed to lower processing losses resulting in higher water content [31]. Higher fat yellowness (b*) in H hams could be an indication of greater lipid oxidation [32]; still, the differences were small, and did not corroborat with TBARS. Salting time only influenced color in BF muscle, with less red (a*), less yellow (b*), and less saturated color (chroma) in hams salted for longer periods. Nevertheless, neither of them had any notable effect on the differences in dry-cured ham color that could be visibly observed (i.e., E*ab greater than 2.0; [33], Table 4).

#### 3.2.3. Texture Measurements of Dry-Cured Hams

The instrumental texture profile was affected by green ham weight but not by salting duration (Table 5). H hams exhibited softer texture characterized by lower hardness in SM, ST, and BF; greater cohesiveness (SM); lower chewiness (ST, BF); higher adhesiveness (SM); and higher Y90 (ST, BF) than L hams, indicating a softer, more plastic texture of the heavy hams, which is in line with a higher moisture content [34,35]. The absence of the salting duration effect differs from the findings of some other studies. For example, Kaltnekar et al. [36] reported that shorter salting durations resulted in softer textures, while Ruiz-Ramírez et al. [37] noted that muscles with lower salt content exhibited reduced hardness, cohesiveness, and springiness. However, in both of the mentioned studies, the applied effects and resulting differences were more pronounced than in our study. Our results on the proteolysis index align with the reported negative correlation between the proteolysis index and hardness [35], i.e., hardness decreased as the proteolysis index increased, particularly in the BF of L hams.

### 3.3. Sensory Analysis of Dry-Cured Hams

Green ham weight or salting duration had a minimal effect on sensory traits (Table 6). Compared to L hams, H ones received lower scores for fresh meat odor and hardness, along with slightly lower sourness scores (*p* < 0.10). The lower hardness evaluated by the expert panel is consistent with instrumentally determined results. Overall, this indicates that H hams had a softer texture, while L hams had a more pronounced fresh meat aroma. As indicated by Buscailhon [3], the higher sourness perception may be related to the higher salt content, which was also observed in the BF muscle of the L hams. However, the sensory panel did not detect differences in saltiness. Regarding the effect of salting time, no difference in perceived saltiness was noted, and the only significant sensorial difference (*p* < 0.05) was determined for sourness. This may again suggest that the perception of sourness is positively related to the salting level or salt content in ham, as previously reported [3]. Regarding the sensory test, it is important to acknowledge a limitation of this study: the model used did not account for variation due to session and assessor, which was instead absorbed within the residual error. Consequently, while this approach may reduce the likelihood of false positive results, it also increases the risk of false negatives.

**Figure 2:**
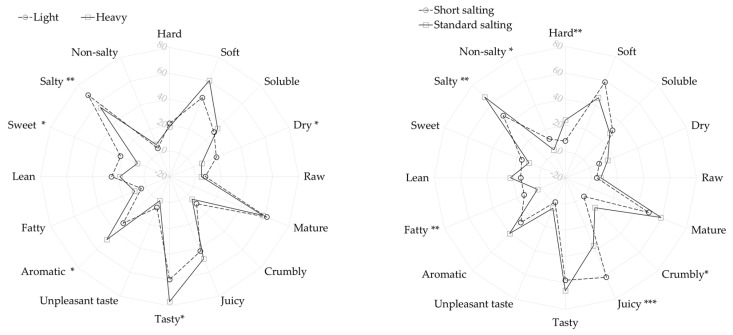
Consumer perceptions as affected by ham weight and salting duration—figures present the frequency (%) of perceived descriptors using the check all that apply (CATA) method. Significance: * = *p* < 0.05; ** = *p* < 0.01; *** = *p* < 0.001.

To obtain a comprehensive view of the tested innovation, sensory testing was also organized with consumers (n = 95) using the CATA test. In the consumer test (Figure 2), H hams were more often described as soft, tasty, and aromatic, while L hams were described as salty, sweet, and dry. This aligns with the observed differences in chemical and rheological characteristics, namely higher salt content, lower moisture levels, and a firmer texture in L hams compared to H hams, as also reported by Andronikov et al. [15]. On the other hand, hams with shorter salting times were more often described as soft, juicy, and fatty, while hams with standard salting times were described as salty and hard, which corroborates with the salt content in the final product. The results indicate that consumers prefer heavier dry-cured hams and shorter salting duration. These results also agree with reports about consumer preferences for Italian and Iberian dry-cured hams [38,39]; traits like high salt content contribute to consumer dislike [40]. It is important to note that a high percentage of consumers (over 60%) described the tasted dry-cured hams as salty, following the order L21 > L14 ≈ H24 > H17. This aligns with the levels of salt content determined chemically, indicating that the reduction in the salting period was insufficient.

## 4. Conclusions

Our study reveals that ham weight and salting duration (2 kg and 7 days difference, respectively) in the studied range showed mild effects on dry-cured hams (mainly on moisture, aw, salt content and texture) traits. Heavier hams were found by the sensory panel to have a softer texture, while longer salting times increased the sourness. We noted a trend of consumer preferences for heavier hams and shorter salting times (i.e., less salty). The applied reduction in the salting phase was insufficient to generate important changes, and the final salt content of the hams remained relatively high. However, by adjusting the salting duration to ham weight, we limited the variability, which is a positive outcome in view of processing optimization. If the industry aims to produce a product that better aligns with consumer preferences for less salty dry-cured hams, the salting phase should be further shortened, and additional optimization in salt reduction should be considered.

## Figures and Tables

**Table 1:** Green ham traits (mean and variation range) according to treatment group.

	L14	L21	H17	H24
Weight, kg	16.6 (15.8–17.2)	16.6 (15.5–17.6)	18.6 (17.9–19.4)	18.5 (17.6–19.1)
Fat thickness, mm	30.1 (17–37)	30.8 (24–39)	33.0 (20–41)	33.6 (25–44)
SM pHu	5.51 (5.40–5.78)	5.53 (5.43–5.82)	5.55 (5.43–5.98)	5.54 (5.42–5.96)

L = lighter hams; H = heavier hams; 14, 17, 21, and 24 denote days of salting; SM pHu = ultimate pH of *semimembranosus* muscle.

**Table 2:** Ham weight losses during processing (at the end of each phase).

	Weight Effect			Salting (Weight) Effect					rmse
	L	H	Sig.	L14	L21	H17	H24	Sig.	
Salting 1	1.7 ^y^	1.5 ^x^	*	1.7	1.8	1.5	1.5	NS	0.22
Salting 2	1.8	1.7	NS	1.4 ^A^	2.1 ^B^	1.2 ^a^	2.3 ^b^	***	0.32
Salting	3.5	3.2	NS	3.1 ^A^	3.9 ^B^	2.6 ^a^	3.8 ^b^	***	0.48
Resting	19.0 ^y^	17.6 ^x^	***	18.7	19.2	17.5	17.8	NS	0.84
Drying	25.8	25.7	NS	25.8	25.7	25.6	25.8	NS	1.01
Ripening	35.7 ^y^	34.1 ^x^	**	35.5	35.9	34.2	34.1	NS	1.29

L = lighter hams; H = heavier hams; 14, 17, 21, and 24 denote days of salting; rmse = root-mean-square error; significance: NS = *p* > 0.10; * = *p* < 0.05; ** = *p* < 0.01; *** = *p* < 0.001. ^x,y^ letters denote significant difference between weight classes (*p* < 0.05). ^A,B^ uppercase letters denote significant difference between salting times within L hams (*p* < 0.05). ^a,b^ lowercase letters denote significant difference between salting times within H hams (*p* < 0.05).

**Table 3:** Chemical traits of dry-cured muscles.

	Weight Effect			Salting (Weight) Effect					
	L	H	Sig.	L14	L21	H17	H24	Sig.	rmse
*Semimembranosus*									
Water, g/kg	407.4 ^x^	437.5 ^y^	**	409.2	405.7	436.1	438.9	NS	22.73
IMF, g/kg	56.7	54.6	NS	56.8	56.5	50.5	58.8	NS	9.89
Protein, g/kg	465.7 ^y^	438 ^x^	**	466.9	464.4	445.2	431.4	NS	23.94
Proteolysis index, %	17.4	17.9	NS	17.6	17.1	17.7	18.1	NS	1.19
Salt, g/kg	60.5	58.5	NS	57.7 ^A^	63.4 ^B^	57.3	59.6	*	3.81
aw	0.846 ^x^	0.862 ^y^	*	0.855	0.837	0.863	0.861	NS	0.0188
TBARS, μg MDA/kg	0.593	0.536	NS	0.566	0.620	0.553	0.520	NS	0.104
*Biceps femoris*									
Water, g/kg	537.1 ^x^	553.6 ^y^	***	543.4	530.7	557.2	550.0	NS	11.35
IMF, g/kg	39.1	40.3	NS	39.0	39.2	40.2	40.4	NS	9.16
Protein, g/kg	315.7 ^y^	305.4 ^x^	**	312.6	318.9	303.9	306.9	NS	8.04
Proteolysis index, %	28.3	27.9	NS	29.1 ^B^	27.5 ^A^	28.0	27.8	*	1.17
Salt, g/kg	84.3 ^y^	79.3 ^x^	*	80.5 ^A^	88.0 ^B^	77.2	81.4	*	5.46
aw	0.857 ^x^	0.875 ^y^	*	0.867	0.847	0.874	0.875	NS	0.0175
TBARS, μg MDA/kg	0.500	0.456	NS	0.495	0.505	0.449	0.463	NS	0.083

L = lighter hams; H = heavier hams; 14, 17, 21, and 24 denote days of salting; rmse = root-mean-square error; significance: NS = *p* > 0.10; * = *p* < 0.05; ** = *p* < 0.01; *** = *p* < 0.001. ^x,y^ letters denote significant difference between weight classes (*p* < 0.05). ^A,B^ uppercase letters denote significant difference between salting times within L hams (*p* < 0.05).

**Table 4:** Color differences (E*ab) of muscles and fat between treatment groups.

	L vs. H	L14 vs. L21	H17 vs. H21
*Semimembranosus*	1.5	1.2	0.7
*Semitendinosus*	1.4	1.6	1.8
*Biceps femoris*	0.6	1.6	1.4
Fat	0.6	1.3	0.7

L = lighter hams; H = heavier hams; 14, 17, 21, and 24 = days of salting.

**Table 5:** Texture measurements of dry-cured ham muscles.

	Weight Effect			Salting (Weight) Effect					
	L	H	Sig.	L14	L21	H17	H24	Sig.	rmse
*Semimembranosus*									
Hardness, N	288.3 ^y^	238.0 ^x^	**	285.8	290.9	229.3	246.7	NS	42.79
Cohesiveness	0.505 ^x^	0.559 ^y^	*	0.512	0.499	0.559	0.559	NS	0.0679
Springiness, mm	5.3	5.6	NS	5.3	5.3	5.6	5.7	NS	0.48
Chewiness, N*mm	755.7	741.0	NS	755.1	756.3	713.5	768.4	NS	123.0
Adhesiveness, N*mm	−0.29 ^x^	−0.48 ^y^	*	−0.29	−0.29	−0.44	−0.52	NS	0.2316
*Semitendinosus*									
Hardness, N	85.9 ^y^	63.1 ^x^	***	83.7	88.1	64.2	62.0	NS	15.60
Cohesiveness	0.589	0.574	NS	0.574	0.604	0.585	0.562	NS	0.0518
Springiness, mm	5.4	5.2	NS	5.2	5.6	5.1	5.3	NS	0.65
Chewiness, N*mm	274.8 ^y^	190.3 ^x^	***	256.6	293.0	194.3	186.3	NS	60.29
Adhesiveness, N*mm	−2.5	−2.5	NS	−2.57	−2.44	−2.67	−2.42	NS	0.8287
Y90	0.604 ^x^	0.616 ^y^	*	0.603	0.605	0.623	0.609	NS	0.0140
*Biceps femoris*									
Hardness, N	111.2 ^y^	91.6 ^x^	**	105.3	117.1	87.8	95.3	NS	18.52
Cohesiveness	0.782	0.758	NS	0.777	0.788	0.777	0.739	NS	0.0369
Springiness, mm	5.3	5.2	NS	5.3	5.4	5.2	5.2	NS	0.42
Chewiness, N*mm	470.0 ^y^	352.8 ^x^	***	441.2	498.9	342.0	363.6	NS	84.64
Adhesiveness, N*mm	−2.05	−2.02	NS	−2.21	−1.88	−1.77	−2.27	NS	0.603
Y90	0.609 ^y^	0.623^x^	*	0.611	0.607	0.631	0.616	NS	0.0173

L = lighter hams; H = heavier hams; 14, 17, 21, and 24 denote days of salting, rmse = root-mean-square error; significance: NS = *p* > 0.10; * = *p* < 0.05; ** = *p* < 0.01; *** = *p* < 0.001. ^x,y^ letters denote significant differences between weight classes (*p* < 0.05).

**Table 6:** Sensory analysis of dry-cured hams.

	Weight Effect			Salting (Weight) Effect					
Whole Slice	L	H	Sig.	L14	L21	H17	H24	Sig.	rmse
Odor									
Fresh meat	3.8 ^y^	3.1 ^x^	*	3.6	3.9	3.3	2.8	NS	0.9
Spicy	2.0	1.8	NS	2.1	1.8	1.7	2.0	NS	0.2
Herbal	1.3	1.2	NS	1.5	1.1	1.0	1.3	NS	0.1
Appearance									
Muscle color	5.6	5.7	NS	5.7	5.5	5.7	5.7	NS	0.3
Fat color	5.8	5.3	NS	5.3	6.3	5.6	5.1	NS	0.3
Color uniformity	3.8	4.0	NS	4.1	3.5	4.2	3.7	NS	0.2
Marbling	4.7	4.5	NS	5.0	4.3	4.4	4.5	NS	0.2
Surface moisture	3.5	3.7	NS	3.9	3.1	3.8	3.6	NS	0.3
Tyrosine crystals	0.14	0.18	NS	0.16	0.13	0.13	0.24	NS	0.3
Taste									
Saltiness	3.7	4.0	NS	3.8	3.6	4.0	3.9	NS	0.4
Sweetness	2.2	2.2	NS	2.1	2.4	2.0	2.4	NS	0
Sourness	1.8	1.5	†	1.4	2.2	1.2	1.7	*	0.5
Bitterness	2.4	2.0	NS	2.1	2.7	1.9	2.1	NS	0.5
Off flavor	0.49	0.47	NS	0.56	0.43	0.35	0.59	NS	0.0
Texture									
Hardness	5.3 ^y^	4.3 ^x^	*	5.0	5.5	4.3	4.3	NS	0.9
Solubility	4.0	4.7	NS	3.6	4.3	4.6	4.9	NS	0.5
Pastiness	3.5	3.6	NS	3.2	3.8	3.3	3.8	NS	0.1

L = lighter hams; H=heavier hams; 14, 17, 21, and 24 = days of salting; rmse = root-mean-square error; significance: NS = *p* > 0.10; † = *p* < 0.10; * = *p* < 0.05. ^x,y^ letters denote significant difference between weight classes (*p* < 0.05).

## Data Availability

The original contributions presented in this study are included in the article/Supplementary Materials. Further inquiries can be directed to the corresponding author.

## Supplementary Materials

The following supporting information can be downloaded at https://www.mdpi.com/article/10.3390/foods13223620/s1. Table S1. Mean value for the overall salt uptake (both salting stages) according to treatment group. Table S2. Calibration parameters of NIR. Table S3. Color measurements of dry-cured ham muscles and fat.
