# Supplementary material for: Dry-Cured Ham, ‘Kraški Pršut’, from Heavy Pig Production—A Pilot Study Focusing on the Effect of Ham Weight and Salting
Source: Foods. 2024 Nov 13;13(22):3620. doi: 10.3390/foods13223620 (PMC11593492; doi:10.3390/foods13223620)
Supplement: Supplementary file 1 [file foods-13-03620-s001.zip › foods-3289321-supplementary.pdf]

Table S1. Mean value for the overall salt uptake (both salting stages) according to treatment group.

	L14	L21	H17	H24
Quantity of applied salt, kg				
Salting 1, kg	0.6	0.6	0.6	0.6
Salting 2, kg	0.4	0.4	0.4	0.4
Total salting, kg	1.0	1.0	1.0	1.0
Salt uptake, kg	0.75	0.75	0.79	0.86
Salt uptake/ kg ham	0.045	0.045	0.042	0.046

L = lighter hams; H = heavier hams; 14,17,21,24 denote days of salting.

Table S2. Calibration parameters of NIR.

N>350	Calibration		Cross-validation		RPD
Constituent	S.E.	R2	S.E.	R2	
Water, g/kg	5.9	0.99	6.3	0.99	9.0
IMF, g/kg	3.9	0.95	4.0	0.95	4.4
Protein, g/kg	6.6	0.99	6.9	0.98	7.8

RPD = residual predictive deviation; IMF = intramuscular fat.

Table S3. Color measurements of dry-cured ham muscles and fat.

	Weight effect			Salting (weight) effect					
	L	H	Sig.	L14	L21	H17	H24	Sig.	rmse
Semimembranosus									
L*	33.6[x]	35.0[y]	**	34.1	33.1	34.7	35.4	NS	1.38
a*	12.6	12.7	NS	12.8	12.4	12.7	12.7	NS	1.02
b*	10.1	9.6	NS	10.3	9.8	9.6	9.5	NS	0.73
Hue	38.6	37.1	NS	39.0	38.2	37.3	37.0	NS	3.11
Chroma	16.2	15.9	NS	16.5	15.9	15.9	15.9	NS	0.92
Semitendinosus									
L*	40.8	41.4	NS	40.7	40.9	40.6	42.2	NS	2.31
a*	12.4	13.4	NS	13.0	11.8	13.2	13.6	NS	1.58
b*	10.3	9.6	NS	10.8	9.8	9.3	9.9	NS	1.11
Hue	39.6[y]	35.8[x]	*	39.7	39.4	35.2	36.4	NS	4.23
Chroma	16.2	16.5	NS	17.0	15.4	16.2	16.9	NS	1.51
Biceps femoris									
L*	38.5	39.1	NS	38.3	38.6	39.0	39.2	NS	1.55
a*	15.9	16.0	NS	16.6[B]	15.3[A]	16.6[b]	15.4[a]	*	1.22
b*	8.0	7.8	NS	8.4[B]	7.6[A]	8.2	7.5	**	0.63
Hue	26.8	26.0	NS	27.1	26.5	26.2	25.9	NS	2.54
Chroma	17.9	17.8	NS	18.6[B]	17.1[A]	18.5[b]	17.1[a]	**	1.13
Fat									
L*	76.6	76.9	NS	77.2	75.9	77.2	76.5	NS	1.30
a*	3.3	3.6	NS	3.2	3.5	3.5	3.7	NS	0.88
b*	7.1[x]	7.5[y]	*	7.0	7.2	7.5	7.5	NS	0.51
Hue	65.3	64.7	NS	66.1	64.5	64.8	64.6	NS	4.83
Chroma	7.9	8.3	NS	7.7	8.0	8.3	8.4	NS	0.76

L = lighter hams; H = heavier hams; 14, 17, 21, 24 denote days of salting;
rmse = root-mean-square error; significance: NS = $p > 0.10$; * = $p < 0.05$; ** = $p < 0.01$; *** = $p < 0.001$.
x,y letters denote significant difference between weight classes ($p < 0.05$).
A,B uppercase letters denote significant difference between salting times within L hams ($p < 0.05$).
a,b lowercase letters denote significant difference between salting times within H hams ($p < 0.05$).
